# Supplementary material for: Investigation of Therapeutic Response Markers for Acupuncture in Parkinson’s Disease: An Exploratory Pilot Study
Source: Diagnostics (Basel). 2021 Sep 17;11(9):1697. doi: 10.3390/diagnostics11091697 (PMC8468821; doi:10.3390/diagnostics11091697)
Supplement: Supplementary file 1 [file diagnostics-11-01697-s001.zip › diagnostics-1368249 - supplementary 1.pdf]

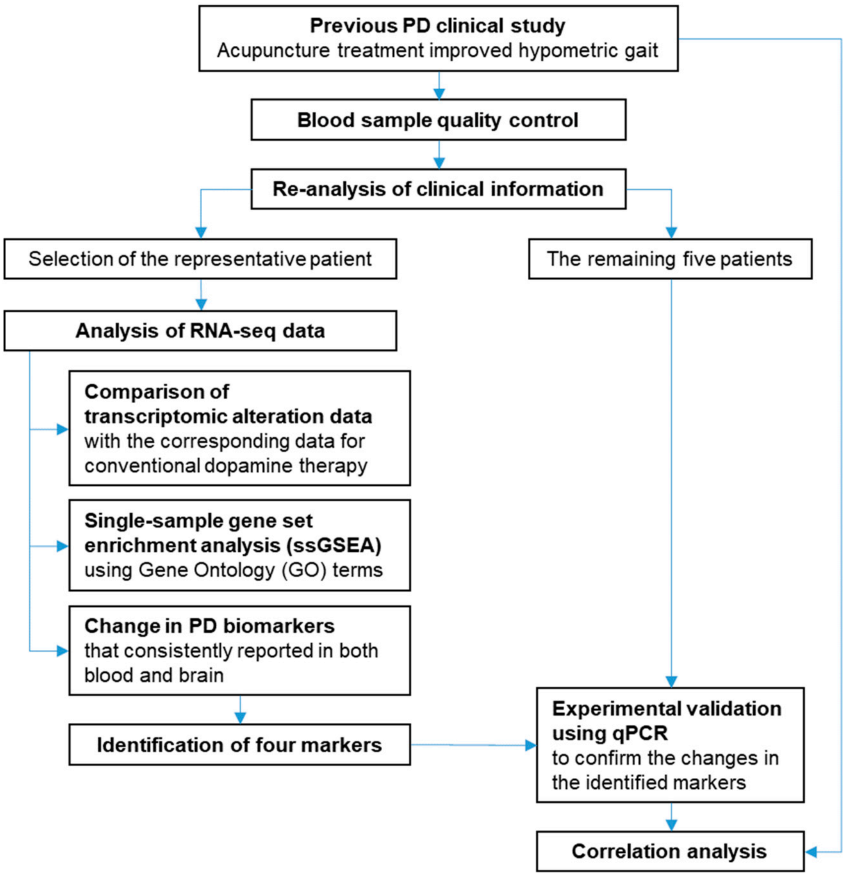

Supplementary Figure S1. Flow diagram of the study.

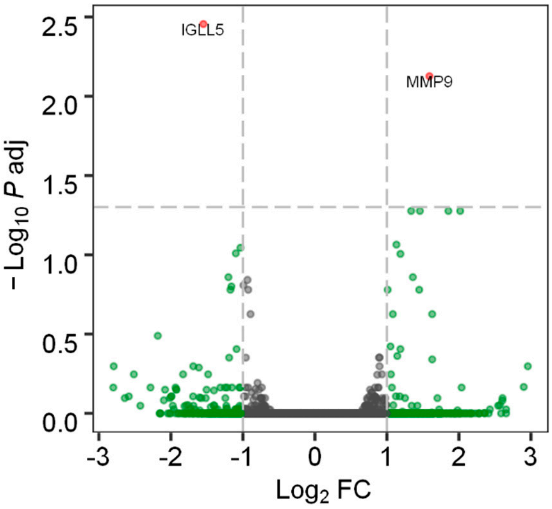

Supplementary Figure S2. Volcano plot for RNA-seq data of the representative patient. Adjusted *P* value (*P*adj) was obtained using Benjamini-Hochberg multiple testing correction from *P*-values. For significance, a false discovery rate of 0.05 was chosen. Due to the small sample size of the treated (V5 and V8; $n = 2$) and untreated (V1; $n = 1$) states, almost all genes were not significant by the thresholds of |fold-change (FC)| > 2 and *P*adj < 0.05.

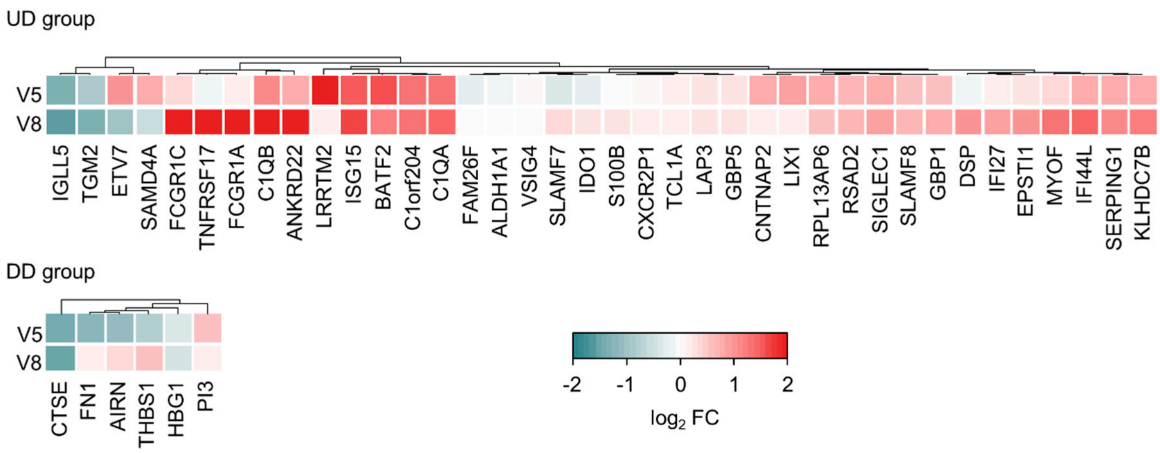

Supplementary Figure S3. Gene expression changes in the UD and DD groups. The 38 upregulated or six downregulated genes after conventional dopaminergic therapy were referred to as UD (upregulated genes by dopaminergic therapy) or DD (downregulated genes by dopaminergic therapy) groups, respectively. Red and blue colors denote increased and decreased expressions, respectively. FC, fold-change.

Abbreviations

Parkinson's disease (PD)
Quantitative real-time polymerase chain reaction (qPCR)
Ankyrin repeat domain 22 (ANKRD22)
Synapsin 1 (SYN1)
Methyl-4-phenyl-1,2,3,6-tetrahydropyridine (MPTP)
Central nervous system (CNS)
RNA sequencing (RNA-Seq)
Prefrontal cortex (PFC)
Supplementary motor area (SMA)
Functional near-infrared spectroscopy (fNIRS)
Gene Ontology (GO)
Biological process (BP)
Single-sample gene set enrichment analysis (ssGSEA)
Inhibitory postsynaptic potential (IPSP)
BTB/POZ domain-containing protein-2 (BPOZ-2)
Differentially expressed gene (DEG)
Alpha-synuclein (α-syn)
S100 calcium-binding protein A10 (p11)
